# Control of Fe^3+^ coordination by excess Cl^−^ in alcohol solutions†

**DOI:** 10.1039/d2ra01522f

**Published:** 2022-06-17

**Authors:** Yunika Nomura, Dai Inoue, Yutaka Moritomo

**Affiliations:** School of Science and Engineering, University of Tsukuba Tsukuba 305-8571 Japan moritomo.yutaka.gf@u.tsukuba.ac.jp; Graduate School of Pure and Applied Sciences, University of Tsukuba Tsukuba 305-8571 Japan; Faculty of Pure and Applied Sciences, University of Tsukuba Tsukuba 305-8571 Japan; Tsukuba Research Center for Energy Materials Science (TREMS), University of Tsukuba Tsukuba 305-8571 Japan

## Abstract

We spectroscopically investigated coordination state of Fe^3+^ in methanol (MeOH) and ethanol (EtOH) solutions against Cl^−^ concentration ([Cl^−^]). In both the system, we observed characteristic absorption bands due to the FeCl_4_ complex at high-[Cl^−^] region. In the MeOH system, the proportion (*r*) of [FeCl_4_]^−^ exhibits a stationary value of 0.2–0.3 in the intermediate region of 10 mM < [Cl^−^] < 50 mM, which is interpretted in terms of [FeCl_*n*_L_6−*n*_]^3−*n*^ (*n* = 1 and 2). In the EtOH system, *r* steeply increases from 0.1 at [Cl^−^] = 1.5 mM to 0.7 at [Cl^−^] = 3.5 mM, indicating direct transformation from [FeL_6_]^3+^ to [FeCl_4_]^−^. We further found that the coordination change significantly decreases the redox potential of Fe^2+^/Fe^3+^.

## Abstract 2

Fe^3+^ coordination in alcohol solution can be controlled by the Cl^−^ concentration ([Cl^−^]). The coordination state changes from FeL_6_ (L: solvent molecule) to FeCl_4_ type *via* FeCl*_n_*L_6−_*_n_* with increases in [Cl^−^].

## Graphical abstract

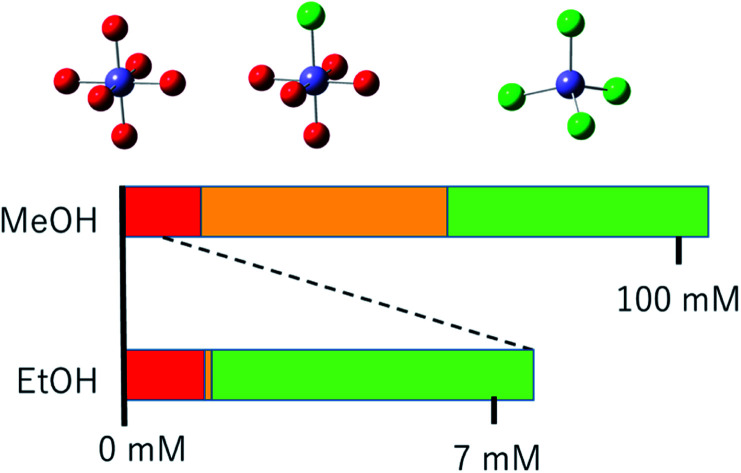

## Introduction

The coordination state around the redox pair in solution has a great influence on the redox potential (*V*) as well as its temperature coefficient (*S*_EC_) because *V* is equivalent to −Δ*G*/*e*, where Δ*G* and *e* are the variation in the Gibbs free energy associated with reduction reaction and elementary charge (>0). In a technological point of view, the electrochemical parameters can be used for energy harvesting device, such as liquid thermoelectric cell (LTE).^1–6^ In this sense, it is scientifically and technologically important to deeply comprehend and control the coordination state of redox pairs in solution. The Fe ion in solution is usually octahedrally coordinated by six solvent molecules (L) forming the FeL_6_ complex. Inada *et al.*^7^ reported that Fe^2+^ is coordinated by six L in aqueous, methanol (MeOH), ethanol (EtOH), dimethyl sulfoxide (DMSO) solutions. If the aqueous solution contains Cl^−^, however, it is reported that Fe^3+^ takes various coordination state,^8,9^ such as [FeCl_*n*_(H_2_O)_6−*n*_]^*n*−3^ and [FeCl_4_]^−^, reflecting a strong interaction between Fe^3+^ and Cl^−^. The Fe coordination in aqueous solution containing Cl^−^ is still controversial.

Recently, there has been an interest in the electrochemistry of redox pairs not only in aqueous solutions but also in organic solutions. Especially, Inoue *et al.*^10^ reported that *S*_EC_ of Fe^2+^/Fe^3+^ in several organic solvents are much higher than *S*_EC_ in aqueous solution. For example, *S*_EC_ (= 3.6 mV K^−10^) of Fe^2+^/Fe^3+^ in acetone is much larger than that (= 1.5 mV K^−6^) in aqueous solution. In addition, Wake *et al.*^11^ reported that the dimensionless performance index (*ZT*) of LTE is much enhanced if aqueous solution of Fe^2+^/Fe^3+^ is replaced with acetone solution of Fe^2+^/Fe^3+^. In this situation, a comprehension of the coordination state of Fe ions in the organic solution is desired. In general, the formation of FeCl_4_ in solution is governed by the equilibrium constant (*K*); *K* = [FeCl_4_^−^]/[Fe^3+^][Cl^−^]^4^ for Fe^3+^ + 4Cl^−^ ↔ FeCl_4_^−^. Then, systematic investigation against Cl^−^ concentration ([Cl^−^]) at a fixed Fe^3+^ concentration is effective for comprehension and control of the complex formation. We emphasized that the ultraviolet-visible (UV-vis) absorption spectroscopy is a sensitive probe for complex formation, because Fe^3+^ complex exhibits characteristic absorption bands in this region.^12^ In addition, the spectroscopy is sensitive even in a dilute Fe^3+^ solution of sub mM and is suitable for investigation of organic solution.

In this work, we spectroscopically investigated coordination state of Fe^3+^ against [Cl^−^] in the MeOH and EtOH solutions. In MeOH solution, with increases in [Cl^−^], octahedral [FeL_6_]^3+^ gradually transforms to tetrahedral [FeCl_4_]^−^*via* mixed [FeCl_*n*_L_6−*n*_]^3−*n*^ state. In EtOH solution, [FeL_6_]^3+^ directly transforms to [FeCl_4_]^−^ with increase in [Cl^−^]. The variation of the Fe coordination significantly decreases *V* of Fe^2+^/Fe^3+^.

## Experiment

### UV-vis absorption

The UV-vis absorption spectra of Fe^3+^ solutions were investigated with a spectrometer (V750, Jasco) at room temperature. Absorption spectra were obtained by dividing the transmission intensity spectra (*I*) of the solution by that (*I*_0_) without cell. The molar absorption coefficient (*ε*) was defined by −ln(*I*/*I*_0_)/*cd*, where *c* (= 0.5 mM) and *d* (= 1 cm) are the molar concentration of Fe^3+^ and thickness of the optical cell, respectively.

The solvents investigated were water (H_2_O), MeOH (FUJIFILM Wako corp.), EtOH (FUJIFILM Wako corp.). The solvents are purchased and used as received. The solutions contain 0.5 mM Fe^3+^. The [Cl^−^] value was controlled by dissolving FeCl_3_·6H_2_O (FUJIFILM Wako corp.), Fe(ClO_4_)_3_·6.9H_2_O (FUJIFILM Wako corp.), and NaCl (FUJIFILM Wako corp.) in appropriate molar ratios. At several [Cl^−^] in MeOH and EtOH solutions, we confirmed that the crystal water in solution does not affect the spectra. The intensity and shape of the spectra of anhydrous FeCl_3_ (FUJIFILM Wako corp.) solutions are essentially the same as that of the corresponding hydrous FeCl_3_ solution. In aqueous solution, LiCl (FUJIFILM Wako corp.) was used instead of NaCl, because LiCl shows higher solubility in water. We further investigated the solutions containing 0.5 mM Fe(ClO_4_)_3_·6.9H_2_O (FUJIFILM Wako corp.). The ClO_4_^−^ concentration ([ClO_4_^−^]) was controlled by dissolving extra NaClO_4_ (FUJIFILM Wako corp.).

### Variation of redox potential *V*

We systematically investigated variation in *V* of Fe^2+^/Fe^3+^ against [Cl^−^]. We define Δ*V* as *V*_sample_ − *V*_ref_, where *V*_sample_ and *V*_ref_ are *V* of the sample and reference cells, respectively. The reference and sample cells were beaker cells which were connected by a salt bridge. The salt bridge was made as follows. NaClO_4_ (10 g per 100 mL) and agar (4 g per 100 mL) were added to water. Then, the solution was heated, dissolved, poured into a U-shaped tube, and cooled to harden. A Pt electrode was inserted into each cell.

The electrolyte in the reference cell was MeOH (or EtOH) solution containing 0.5 mM Fe(ClO_4_)_2_·6H_2_O (FUJIFILM Wako corp.) and 0.5 mM Fe(ClO_4_)_3_·6.9H_2_O. The electrolytes in the sample cell were [Cl^−^] controlled MeOH (or EtOH) solution containing 0.5 mM Fe^2+^ and 0.5 mM Fe^3+^. The [Cl^−^] value was controlled by dissolving FeCl_2_·4H_2_O, FeCl_3_·6H_2_O, Fe(ClO_4_)_2_·6H_2_O, Fe(ClO_4_)_3_·6.9H_2_O, and NaCl in appropriate molar ratios. Δ*V* between the cells were carefully investigated with confirming stabilization of the voltage. At several [Cl^−^] in MeOH and EtOH solutions, we confirmed that the crystal water in solution does not affect the redox potential. The redox potentials of anhydrous FeCl_2_ (FUJIFILM Wako corp.)/FeCl_3_ (nacalai tesque) solutions are the same as that of the corresponding hydrous FeCl_2_/FeCl_3_ solutions within the experimental error (<6 mV).

## Results and discussion

### Overall feature of spectra

**Fig. 1:**
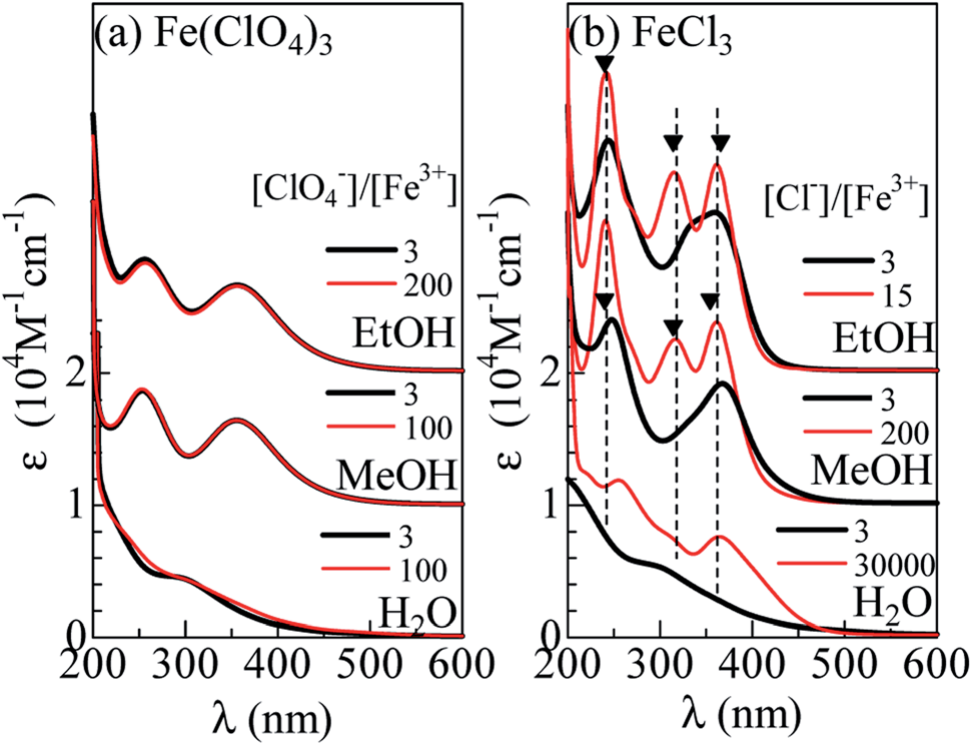
Molar absorption coefficient (*ε*) spectra of (a) Fe(ClO_4_)_3_ and (b) FeCl_3_ dissolved in H_2_O, methanol (MeOH) and ethanol (EtOH). Thin curves (a) and (b) are the spectra with excess ClO_4_^−^ and Cl^−^, respectively. Filled triangles in (b) represent the absorption bands due to [FeCl_4_]^−^.

Fig. 1(a) shows molar absorption coefficient (*ε*) spectra of Fe(ClO_4_)_3_ dissolved in H_2_O, MeOH, and EtOH. Thick and thin curves represent for the spectra without and with excess ClO_4_^−^, respectively. In all solutions, the spectra with excess ClO_4_^−^ are the same as those without excess ClO_4_^−^. This observation indicates that the Fe^3+^ complex is stable even with excess ClO_4_^−^. In MeOH and EtOH solutions, the spectra exhibit two absorption bands at 360 nm and 260 nm. We ascribed the spectral feature to formation of [FeL_6_]^3+^. We calculated the absorption spectra of [Fe(MeOH)_6_]^3+^ cluster with Gaussian 16W program^13^ (Fig. S1†). The calculated spectrum shows two-band structure at 190 nm and 370 nm due to the ligand to metal charge transfer (LMCT) transition and qualitatively reproduces the observed spectra [Fig. 1(a)]. In aqueous solution, traces of absorption bands are discernible at 300 nm, which is ascribed to [Fe(OH)(H_2_O)_5_]^2+^.^14,15^

Fig. 1(b) shows the *ε* spectra of FeCl_3_ dissolved in H_2_O, MeOH, and EtOH. Thick curves represent for the spectra without excess Cl^−^. The spectrum of the MeOH and EtOH solutions without excess Cl^−^ still exhibit two-band structure at 250 nm and 370 nm. The spectral weight of the 260 nm band is higher than that of the Fe(ClO_4_)_3_ solutions [(*a*)]. We ascribed the spectral feature to formation of [FeCl_*n*_L_6−*n*_]^3−*n*^ (*n* = 1 and 2), because the oscillator strength (*f*) of an electron transfer from Cl to Fe^3+^ is larger than that from O to Fe^3+^. We calculated the absorption spectra of [FeClMeOH_5_]^2+^, *trans* [FeCl_2_MeOH_4_]^+^, and *cis* [FeCl_2_MeOH_4_]^+^ clusters with Gaussian 16W program^13^ (Fig. S1†). The total oscillator strength (*f*_tot_) of transitions forming the higher energy band is larger in the Cl-substituted clusters than *f*_tot_ (= 0.42) in [FeMeOH_6_]^3+^; *f*_tot_ is 0.56 in [FeClMeOH_5_]^2+^, 0.73 in *trans* [FeCl_2_MeOH_4_]^+^, and 0.74 in *cis* [FeCl_2_MeOH_4_]^+^.The spectral shape of the FeCl_3_ aqueous solution without excess Cl^−^ is the same as that of the Fe(ClO_4_)_3_ aqueous solution, indicating that the Fe coordination state remains [Fe(OH)(H_2_O)_5_]^2+^.^14,15^

**Fig. 2:**
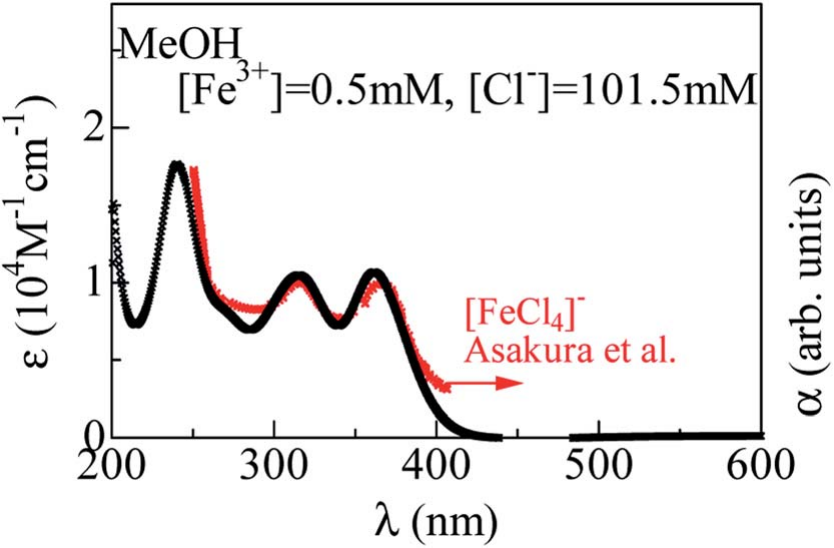
Molar absorption coefficient (*ε*) spectra of MeOH solution containing 0.5 mM Fe^3+^ at [Cl^−^] = 101.5 mM. Red symbols show the *ε* spectra of nitromethane solution of [N(C_2_H_5_)_4_][FeCl_4_] (cited from ref. 8).

Surprisingly, addition of excess Cl^−^ completely changes the spectra. Thin curves in Fig. 1(b) represent for the spectra with excess Cl^−^. In the MeOH and EtOH solutions, excess Cl^−^ causes sharp absorption bands at 362, 318, and 242 nm, as indicated by filled triangles. We emphasize that the spectral profile of the MeOH solution is the same as that of the EtOH solution. The sameness of the two spectra indicates that Fe^3+^ is not coordinated by L, but Cl^−^. In Fig. 2, we compared the spectra of the MeOH solution containing 0.5 mM Fe^3+^ with excess at [Cl^−^] = 101.5 mM with that of nitromethane solution of [N(C_2_H_5_)_4_][FeCl_4_].^3^ Concerning to the lower-lying two absorption bands, the spectral profiles are essentially the same. This clearly indicates formation of [FeCl_4_]^−^ in the MeOH and EtOH solutions with excess Cl^−^. On the other hands, the spectrum of aqueous solution at [Cl^−^]/[Fe^3+^] = 30 000 exhibits two-band structure. The spectral profile is consistent with the spectra at [Cl^−^] = 15 M reported by Liu *et al.*,^12^ who ascribed the spectral feature to [FeCl_3_(H_2_O)_3_] and/or [FeCl_4_]^−^. We emphasize that the 260 nm band is located at higher wavelength side than the 242 nm band due to [FeCl_4_]^−^, as indicated by broken straight lines. This clearly indicates that [FeCl_4_]^−^ is not the dominant species even at the highest [Cl^−^].

### Spectral change against [Cl^−^]

**Fig. 3:**
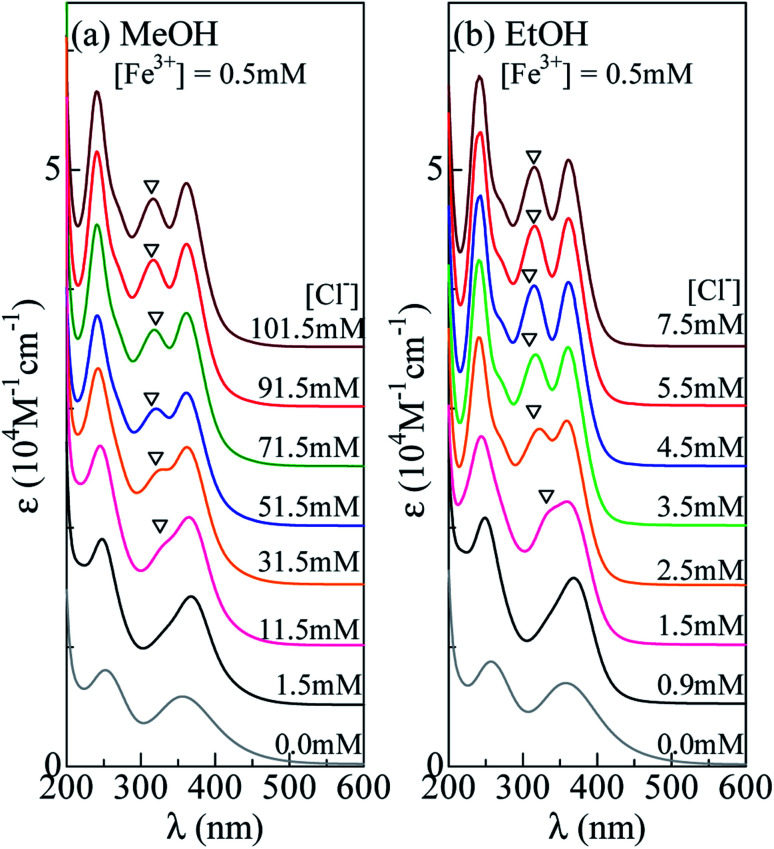
Molar absorption coefficient (*ε*) spectra of (a) MeOH and (b) EtOH solution containing 0.5 mM Fe^3+^ against Cl^−^ concentration ([Cl^−^]). Open triangles indicate FeCl_4_ band (see text).

Now, let us investigate in detail how the *ε* spectrum changes with increase in [Cl^−^]. Fig. 3(a) shows the *ε* spectra of the MeOH solution containing 0.5 mM Fe^3+^ against [Cl^−^]. At [Cl^−^] = 0.0 mM, the spectrum exhibits two broad absorption bands at 360 nm and 260 nm, which are due to an electron transfer from L to Fe^3+^ within the FeL_6_ complex. At [Cl^−^] = 1.5 mM, the spectra still show two-band structure, but the spectral weight of the higher energy band is much higher than those at [Cl^−^] = 0.0 mM. As discussed in the previous subsection, the spectral change is interpreted in terms of the formation of [FeCl_*n*_L_6−*n*_]^3−*n*^ (*n* = 1 and 2). At [Cl^−^] = 11.5 mM, trace of an additional absorption band is discernible, as indicated by an open triangle. Its spectral weight gradually increases as [Cl^−^] increases. At [Cl^−^] = 71.5 mM, the spectrum shows characteristic three band structure due to FeCl_4_^−^ (Fig. 2). The spectra remain unchanged in the [Cl^−^] region above 71.5 mM. This means that all Fe^3+^ form the [FeCl_4_]^−^ complex, because the equilibrium does not move with increase in [Cl^−^]. Hereafter, we will call the absorption band at 318 nm as “FeCl_4_ band”. A detailed investigation revealed three isosbestic points at 225 nm, 255 nm, and 385 nm above [Cl^−^] = 11.5 mM [Fig. S2(a)†]. The isosbestic point reflects the reaction between octahedral ([FeCl_*n*_L_6−*n*_]^3−*n*^) to tetrahedral ([FeCl_4_]^−^) complexes with increase in [Cl^−^].

A similar [Cl^−^]-dependent spectral change is observed in the EtOH solution [Fig. 3(b)]. At [Cl^−^] = 0.0 and 0.9 mM, the spectra exhibit two-band structure. At [Cl^−^] = 2.5 mM, an additional absorption band appears as indicated by an open triangle. Its spectral weight steeply increases as [Cl^−^] increases. At [Cl^−^] = 4.5 mM, the spectrum shows characteristic three band structure due to FeCl_4_ (Fig. 2). The spectra remain unchanged in the [Cl^−^] region above 4.5 mM, indicating that all Fe^3+^ forms the FeCl_4_ complex. We found three isosbestic points at 225 nm, 260 nm, and 385 nm above [Cl^−^] = 0.9 mM [Fig. S2(b)†]. The isosbestic point reflects the reaction between octahedral ([FeCl_*n*_L_6−*n*_]^3−*n*^) to tetrahedral ([FeCl_4_]^−^) complexes with increase in [Cl^−^].

### FeCl_4_ formation against [Cl^−^]

The solution system investigated contains multiple complex species, such as, [FeL_6_]^3+^, [FeCl_*n*_L_6−*n*_]^3−*n*^ (*n* = 1 and 2), [FeCl_4_]^−^. It is difficult to unambiguously decompose the spectrum into the respective components because there is no quantitative information on the spectra due to [FeCl_*n*_L_6−*n*_]^3−*n*^. Fortunately, we know the spectrum of [Fe^III^Cl_4_]^−^. In addition, the FeCl_4_ band at 318 nm is well separated from the absorption bands due to other complexes. In the following, we focused our attention on the FeCl_4_ formation against [Cl^−^]. We evaluated the intensities and peak positions (*λ*_p_) of the FeCl_4_ band by least-squares fitting with three Gaussian functions (Fig. S3 and S4†). The intensities were normalized by the value at [Cl^−^] = 101.5 mM (7.5 mM) for the MeOH (EtOH) solutions, where all Fe^3+^ is considered to form the FeCl_4_ complex. Then, the proportion (*r*) of the FeCl_4_ complex is the same value as the normalized intensity (*I*).

**Fig. 4:**
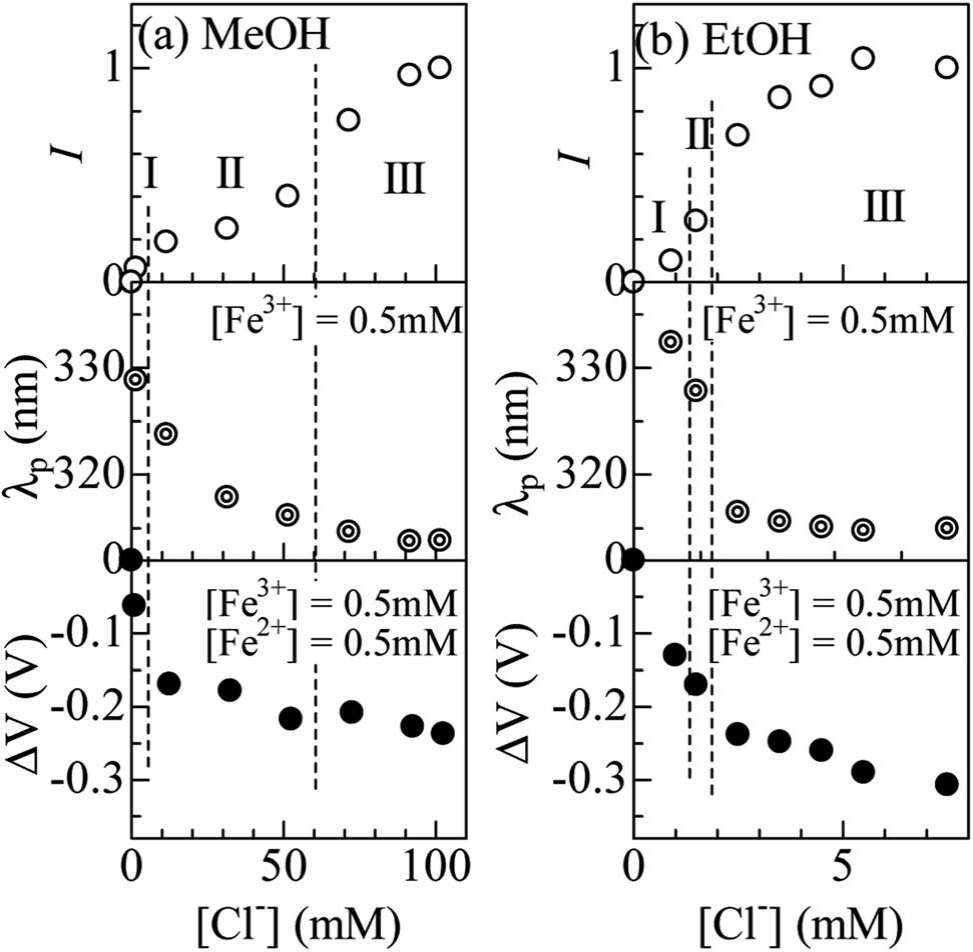
(a) Normalized intensity (*I*; upper panel) and wavelength (*λ*_p_; middle panel) at the peak of the FeCl_4_ band, and variation (Δ*V*; bottom panel) in *V* of Fe^2+^/Fe^3+^ in MeOH solution against [Cl^−^]. The intensity was normalized by the value at [Cl^−^] = 101.5 mM. (b) *I* (upper panel) and *λ*_p_ (middle penal), and Δ*V* (bottom panel) in EtOH solution against [Cl^−^]. The intensity was normalized by the value at [Cl^−^] = 7.5 mM. For convenience of explanation, regions I, II, and III are defined (see text).

Fig. 4 shows *I* (upper panel) and *λ*_p_ (middle panel) against [Cl^−^] in (a) MeOH and (b) EtOH solutions. For convenience of explanation, we define regions I, II, and III as regions where *r* (= *I*) < 0.2, 0.2 < *r* < 0.7, and *r* > 0.7, respectively. The region I and III are dominated by the [FeL_6_]^3+^ and [FeCl_4_]^−^ complexes, respectively. In (a) MeOH system, *r* exhibits a stationary value of 0.2–0.3 in the region of 10 mM < [Cl^−^] < 50 mM (region II). The fact that *r* remains 0.2–0.3 indicates existence of a dominant species other than [FeCl_4_]^−^ and [FeL_6_]^3+^, that is, [FeCl_*n*_L_6−*n*_]^3−*n*^ (*n* = 1 and 2). In (b) EtOH, *r* steeply increases from 0.5 at [Cl^−^] = 1 mM to 0.7 at [Cl^−^] = 2.5 mM. The steep increase in *r* indicates that FeL_6_ almost directly transfers to FeCl_4_ with increases in [Cl^−^].

The difference in the complex formation between the MeOH and EtOH solutions is probably reflects the difference in solubility of Cl^−^. The solubility (= 6 mM) of NaCl in EtOH is much smaller than that (= 100 mM) in MeOH. In EtOH system, Cl^−^ in solution is unstable and tend to form the [FeCl_4_]^−^ complex if Fe^3+^ exits in solvent. The [Cl^−^] value (= 2.0 mM) at the boundary to region III is nearly the same value (= 2.0 mM) as required for all Fe^3+^ to become FeCl_4_. In MeOH system, Cl^−^ in solution is stabler than in EtOH. This causes the stable [FeL_6_]^3+^ in small [Cl^−^] region (<10 mM; region I) and [FeCl_*n*_L_6−*n*_]^3−*n*^ formation in the intermediate [Cl^−^] region (10 mM < [Cl^−^] < 50 mM; region II). In aqueous solution where Cl^−^ is much more stabler than in alcohol solutions, no trace of [FeCl_4_]^−^ is observed even at [Cl^−^] = 15 M [Fig. 1(b)]. Thus, our investigation revealed the solvent dependence in complex formation against [Cl^−^].

### Coordination effect on *V*

Now, let us investigate the correlation between the Fe ion coordination and *V* of Fe^2+^/Fe^3+^. The bottom panel of Fig. 4 shows Δ*V* (= *V*_sample_ − *V*_ref_) of Fe^2+^/Fe^3+^ in (a) MeOH and (b) EtOH solutions. The electrolyte in the reference cell was MeOH (or EtOH) solution containing 0.5 mM Fe^2+^ and 0.5 mM Fe^3+^ at [Cl^−^] = 0.0 mM, where the FeL_6_ complex is dominated. We must confess that we have no detailed information on the complex state of Fe^2+^ against [Cl^−^]. Like Fe^3+^, the complex state is considered to change from [FeL_6_]^2+^, [FeCl_*n*_L_6−*n*_]^2−*n*^, to [FeCl_4_]^2−^ with increase in [Cl^−^]. In the following argument, we assume that the redox reaction of the complex occurs without substitution of the coordinated molecules/ions.

In (a) MeOH system, Δ*V* is −0.16 V at the entrance of region II. The variation in *V* between [FeL_6_]^2+^/[FeL_6_]^3+^ (region I) and [FeCl_*n*_L_6−*n*_]^2−*n*^/[FeCl_*n*_L_6−*n*_]^3−*n*^ (region II) is well explained in terms of the crystal field splitting. We note that six d electrons in the octahedral [FeL_6_]^2+^ and [FeCl_*n*_L_6−*n*_]^2−*n*^ takes high-spin (HS) configuration, similarly to the case of [Fe(H_2_O)_6_]^2+^.^16,17^ The crystal field from Cl^−^ is weaker than that from oxygen in L. Then, *V* of [FeCl_4_]^2−^/[FeCl_4_]^−^ is expected to be lower, reflecting narrower crystal field splitting. In (b) EtOH system, Δ*V* is −0.24 *V* at the entrance of region III. Variation of *V* between [FeL_6_]^2+^/[FeL_6_]^3+^ (region I) and [FeCl_4_]^2−^/[FeCl_4_]^−^ (region III) is also explained in terms of the crystal filed splitting. We note that six d electrons take the HS configuration in tetrahedral [FeCl_4_]^2−^, because the crystal field splitting (= 10 Dq) in octahedral complex is larger than that (= 4.45 Dq) in the tetrahedral complex.^18^*V* of [FeCl_4_]^2−^/[FeCl_4_]^−^ is expected to be lower, reflecting narrower ligand field splitting.

## Conclusions

We spectroscopically investigated variation of the Fe ion coordination against [Cl^−^] in MeOH and EtOH solutions. In MeOH solution, with increases in [Cl^−^], octahedral [FeL_6_]^3+^ gradually transforms to tetrahedral [FeCl_4_]^−^*via* mixed [FeCl_*n*_L_6−*n*_]^3−*n*^ (*n* = 1 and 2). In EtOH solution, [FeL_6_]^3+^ directly transforms to [FeCl_4_]^−^ with increase in [Cl^−^]. We found that the coordination change significantly decreases *V* of Fe^2+^/Fe^3+^ and interpreted the observation in terms of the ligand field splitting.

## Author contributions

Y. N. performed spectroscopic and electrochemical experiment. D. I. have supported the experiment and analysis. D. I further performed quantum chemistry calculation. Y. M. made the experimental plan and wrote the manuscript.

## Conflicts of interest

There are no conflicts to declare.

## Supplementary Material

RA-012-D2RA01522F-s001
