# Supplementary material for: Control of Fe3+ coordination by excess Cl− in alcohol solutions
Source: RSC Adv. 2022 Jun 17;12(28):17932–6. doi: 10.1039/d2ra01522f (PMC9204708; doi:10.1039/d2ra01522f)
Supplement: RA-012-D2RA01522F-s001 [file RA-012-D2RA01522F-s001.pdf]

Electronic Supplementary Material (ESI) for RSC Advances.
This journal is © The Royal Society of Chemistry 2022

Electronic Supporting information

Control of Fe^{3+} coordination by excess Cl^- in alcohol solutions

Yunika Nomura,[a] Dai Inoue[b] and Yutaka Moritomo*[a,b,c,d]

a School of Science and Engineering, University of Tsukuba, Tsukuba, 305-8571, Japan

b Graduate School of Pure ad Applied Science, University of Tsukuba, Tsukuba, 305-8571, Japan

c Faculty of Pure and Applied Science, University of Tsukuba, Tsukuba, 305-8571, Japan

d Tsukuba Research Center for Energy Materials Science (TREMS), University of Tsukuba, Tsukuba, 305-8571, Japan

Contact information:

Yutaka Moritomo

Faculty of Pure and Applied Sciences, and

Tsukuba Research Center for Energy Materials Science (TREMS)

Univ. of Tsukuba, Tennodai 1-1-1, Tsukuba 305-8571, Japan

Tel & Fax +81-29-853-4337

e-mail: moritomo.yutaka.gf@u.tsukuba.ac.jp

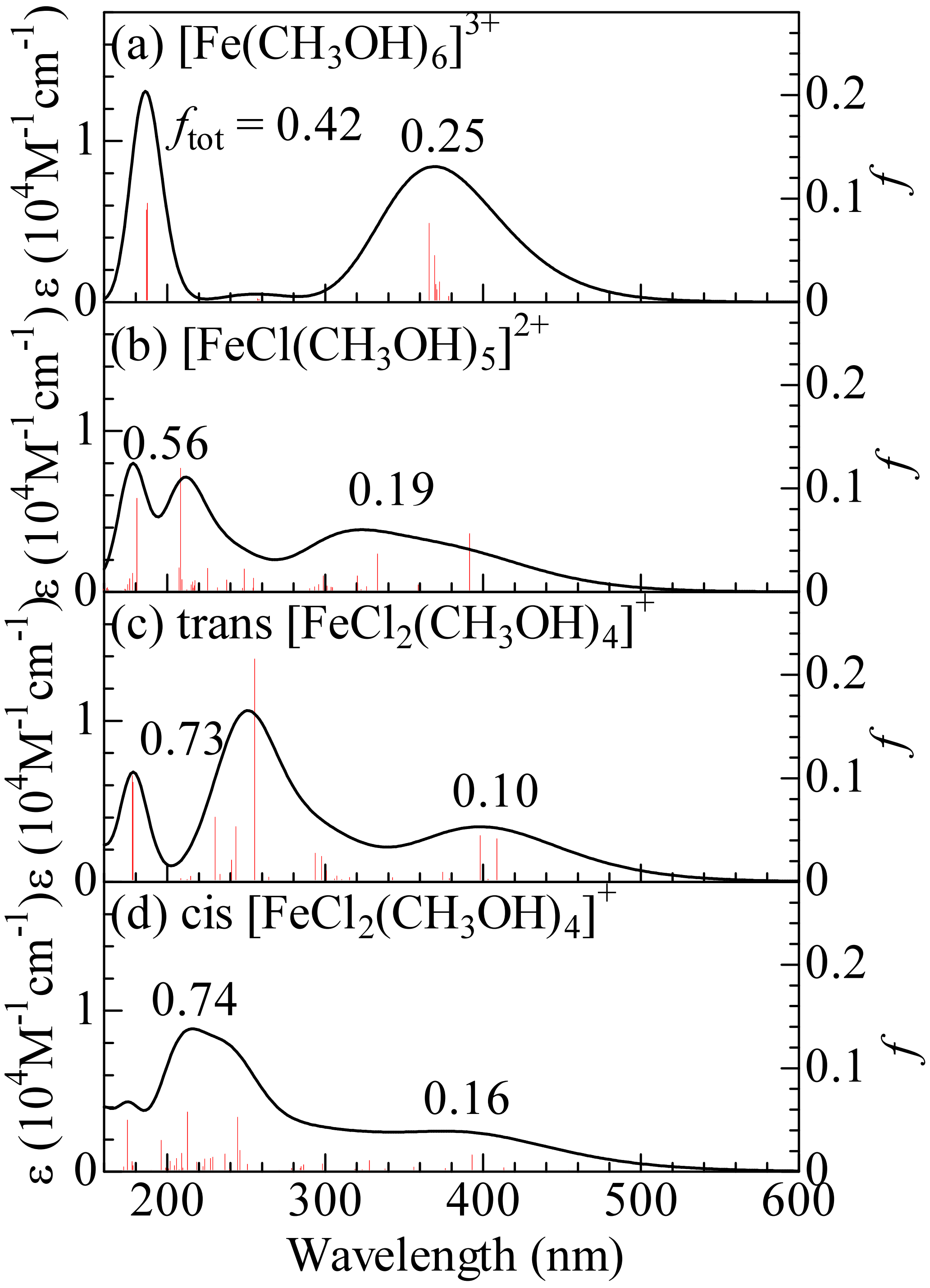

Fig S1. Calculated molar absorption coefficient (ε) spectra of (a) $[Fe(CH_3OH)_6]^{3+}$, (b) $[FeCl(CH_3OH)_5]^{2+}$, (c) trans $[FeCl_2(CH_3OH)_4]^{+}$, and (d) cis $[FeCl_2(CH_3OH)_4]^{+}$ clusters. The vertical bars represent oscillator strength (f). The spectra were calculated with a full width at half maximum of 0.4 eV. f_{tot} is the total oscillator strength of transitions forming

the band. Calculations were performed with Gaussian 16W program[1] at the UwB97XD/6-311+G(d,p) level. Solvation effect of CH_3OH was included by integral equation formalism-polarizable continuum model (IEF-PCM[2]). After the structural optimization, the excited states were calculated within the framework of time-dependent density-functional theory (TD-DFT[3]).

1. Gaussian 16, Revision A.03, M. J. Frisch, G. W. Trucks, H. B. Schlegel, G. E. Scuseria, M. M. A. Robb, J. R. Cheeseman, G. Scalmani, V. Barone, G. A. Petersson, H. Nakatsuji, X. Li, M. Caricato, A. V. Marenich, J. Bloino, B. G. Janesko, R. Gomperts, B. Mennucci, H. P. Hratchian, J. V. Ortiz, A. F. Izmaylov, J. L. Sonnenberg, D. Williams-Young, F. Ding, F. Lipparini, F. Egidi, J. Goings, B. Peng, A. Petrone, T. Henderson, D. Ranasinghe, V. G. Zakrzewski, J. Gao, N. Rega, G. Zheng, W. Liang, M. Hada, M. Ehara, K. Toyota, R. Fukuda, J. Hasegawa, M. Ishida, T. Nakajima, Y. Honda, O. Kitao, H. Nakai, T. Vreven, K. Throssell, J. A. Montgomery, Jr., J. E. Peralta, F. Ogliaro, M. J. Bearpark, J. J. Heyd, E. N. Brothers, K. N. Kudin, V. N. Staroverov, T. A. Keith, R. Kobayashi, J. Normand, K. Raghavachari, A. P. Rendell, J. C. Burant, S. S. Iyengar, J. Tomasi, M. Cossi, J. M. Millam, M. Klene, C. Adamo, R. Cammi, J. W. Ochterski, R. L. Martin, K. Morokuma, O. Farkas, J. B. Foresman and D. J. Fox, Gaussian, Inc., Wallingford CT, 2016.
2. M. Cossi, G. Scalmani, N. Rega, and V. Barone, J. Chem. Phys., 43, **117**, 2002; M. T. Cancès, B. Mennucci, and J. Tomasi, J. Chem. Phys., 3032, **107**, 1997; M. Cossi, V. Barone, B. Mennucci, and J. Tomasi, Chem. Phys. Lett., 253, **286**, 1998; B. Mennucci and J. Tomasi, J. Chem. Phys., 5151, **106**, 1997.
3. R. Erich, and Gross, E. K. U, Phys. Rev. Lett., 997, **52**, 1984.

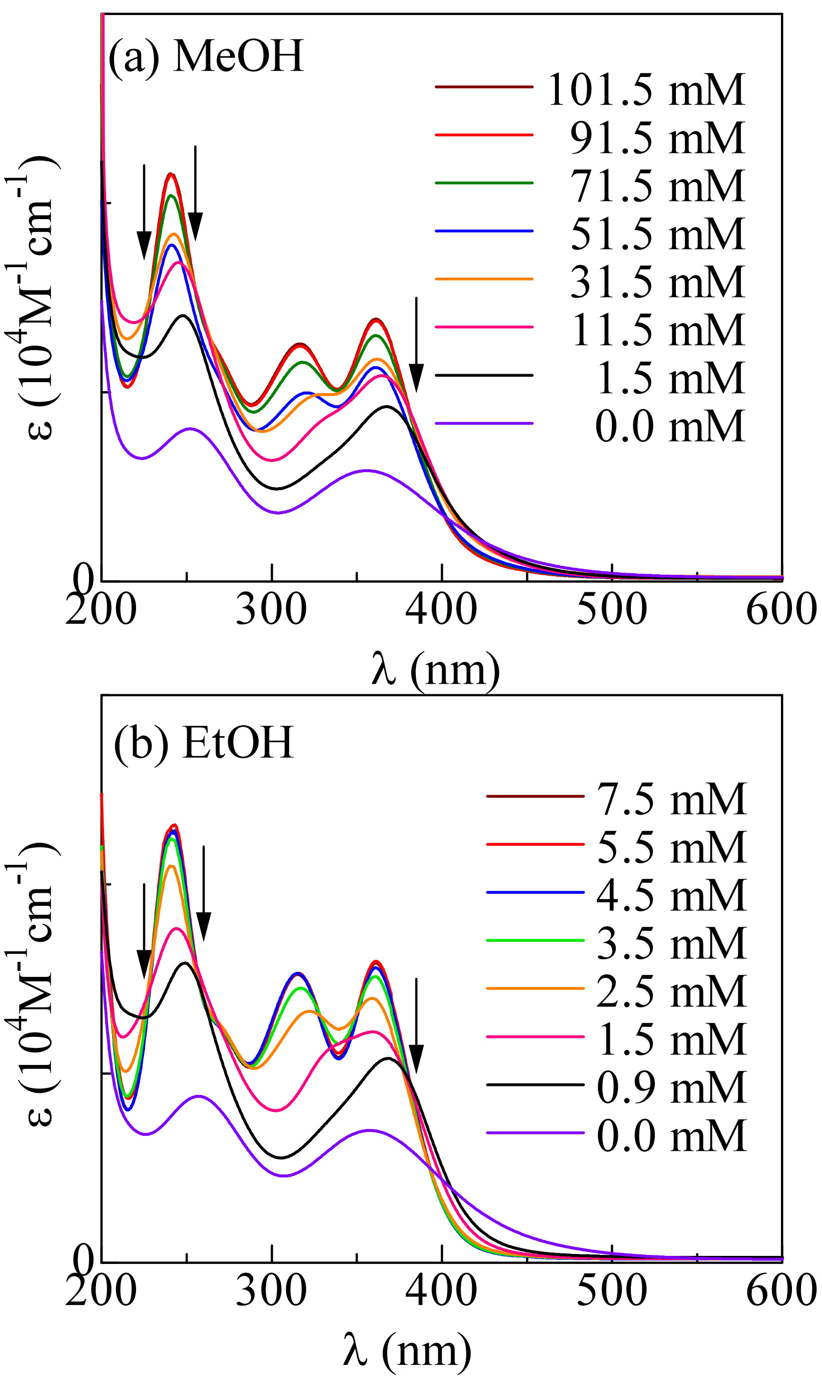

Fig. S2: Molar absorption coefficient (ε) spectra of (a) MeOH and (b) EtOH solution containing 0.5 mM Fe^{3+} against Cl^- concentration ([Cl^-]). Arrows indicate isosbestic points

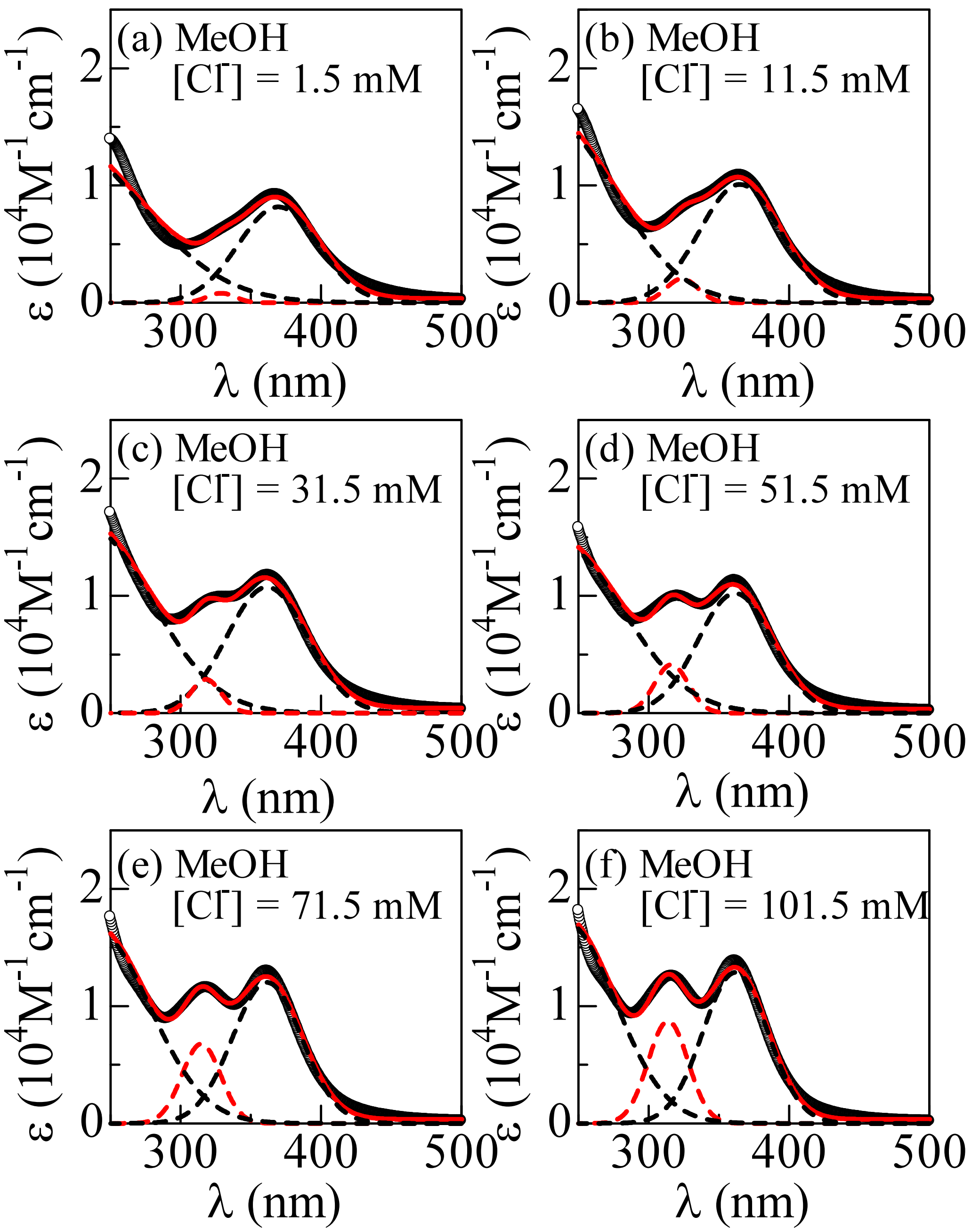

Fig. S3: Molar absorption coefficient (ε) spectra of the MeOH solutions containing 0.5 mM Fe^{3+}: (a) [Cl^-] = 1.5 mM, (b) 11.5 mM, (c) 31.5 mM, (d) 51.5 mM, (e) 71.5 mM, and (f) 101.5 mM. Broken curves represent results of least-squares fitting with three Gauss functions. The component at 318 nm is the $FeCl_4$ band.

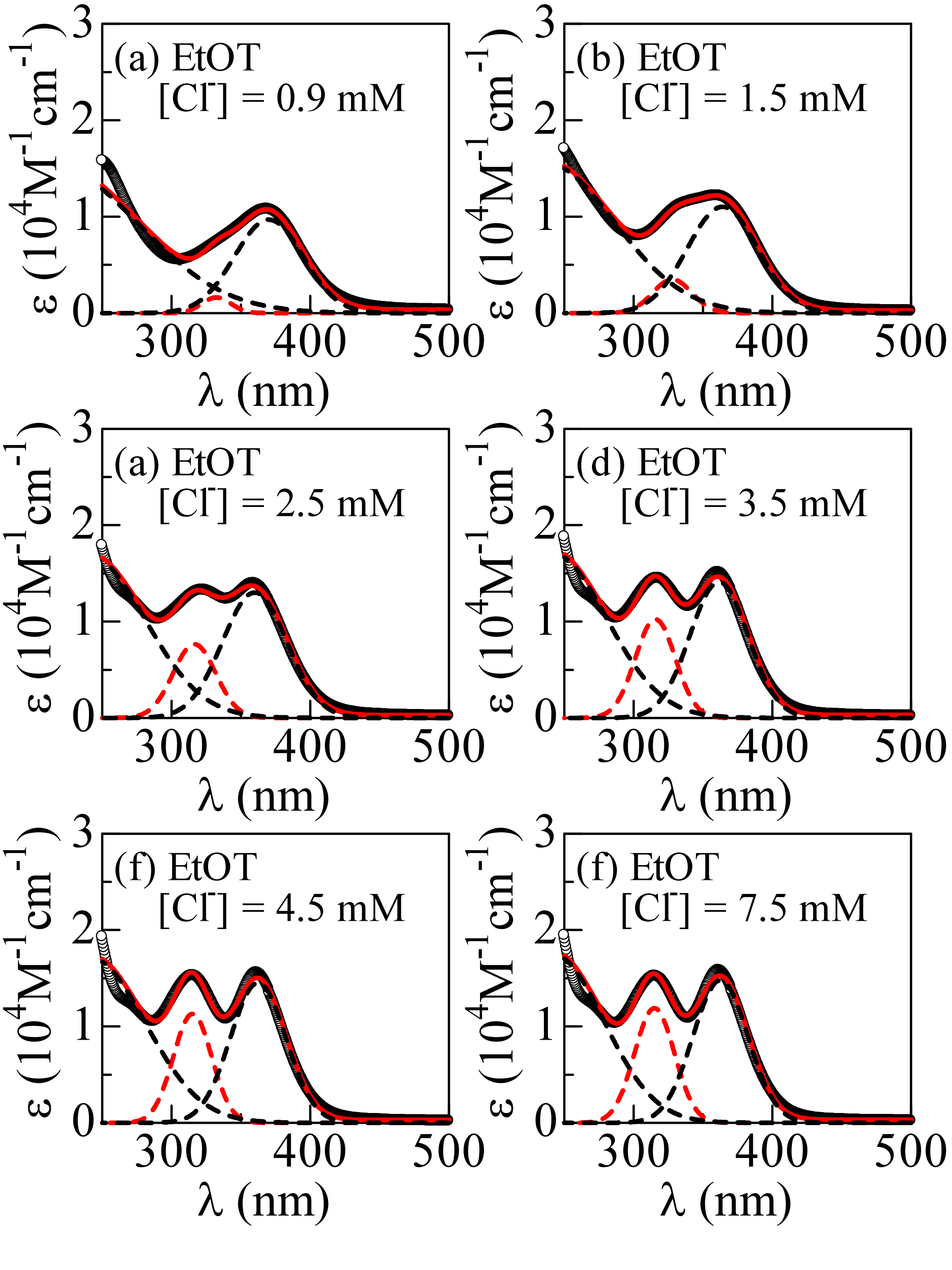

Fig. S4: Molar absorption coefficient (ε) spectra of the EtOH solutions containing 0.5 mM Fe^{3+}: (a) [Cl^-] = 0.9 mM, (b) 1.5 mM, (c) 2.5 mM, (d) 3.5 mM, (e) 4.5 mM, and (f) 7.5 mM. Broken curves represent results of least-squares fitting with three Gauss functions. The component at 318 nm is the $FeCl_4$ band.
